# CD81 promotes proliferation and predicts survival in lung squamous cell carcinoma

**DOI:** 10.1002/ctm2.70672

**Published:** 2026-04-20

**Authors:** Ryu Kanzaki, Steven Reid, Paulina Bolivar, Sara Larsson, Yasushi Shintani, Kristian Pietras

**Affiliations:** ^1^ Division of Translational Cancer Research Department of Laboratory Medicine Lund University Cancer Centre Lund University Lund Sweden; ^2^ Department of General Thoracic Surgery Osaka University Graduate School of Medicine Suita Japan; ^3^ Department of General Thoracic Surgery Osaka International Cancer Institute Osaka Japan

**Keywords:** CD81, lung squamous cell carcinoma

1

Dear Editor,

We demonstrated that CD81 promotes tumour growth in lung squamous cell carcinoma (LUSC) and serves as a novel adverse prognostic marker in resected cases. Although the role of CD81 in LUSC has been previously suggested based on bioinformatics analyses, we validated its tumour‐promoting function using analyses of clinical specimens and experimental models.

LUSC remains a challenging non‐small‐cell lung cancer (NSCLC) subtype due to the lack of established molecular targets and prognostic biomarkers, underscoring the need for novel markers to guide therapy. Previous studies have demonstrated that CD81 plays context‐dependent roles in cancer biology, acting as a tumour promoter in some malignancies (e.g., melanoma, breast and prostate cancer) and as a tumour suppressor in others (e.g., hepatocellular carcinoma and bladder cancer).^1^ In the context of NSCLC, recent bioinformatic analyses suggest that CD81 expression may be associated with adverse outcomes in LUSC, whereas it may have protective effects in adenocarcinoma.^2^ However, these findings have not been validated in resected tumour tissues or sufficiently examined in experimental models.

Materials and methods of the present study are shown in the Supporting Information S1. We examined CD81 expression in surgically resected LUSC tissues from 101 patients (Table S1). Immunohistochemistry revealed CD81 expression in both tumoural and stromal compartments. Given the distinct biological roles of tumour and stromal compartments,^3^ analyses were performed separately, with a primary focus on tumoural CD81 expression. Thirty‐six percent of tumours exhibited positive CD81 staining in cancer cells, whereas stromal CD81 expression was observed in 19%. Patients with CD81‐positive tumours had significantly worse 5‐year relapse‐free survival (42% vs. 64%, *p* = .0066) and overall survival (50% vs. 68%, *p* = .0286) compared with those with CD81‐negative tumours, while stromal CD81 expression was not associated with outcomes (Figure 1). Multivariate Cox regression analyses identified tumoural CD81 expression, elevated preoperative CYFRA21‐1 serum levels, and restrictive ventilatory impairment (%VC < 80%) as independent prognostic indicators of worse overall survival (Tables S2 and S3).

**FIGURE 1:**
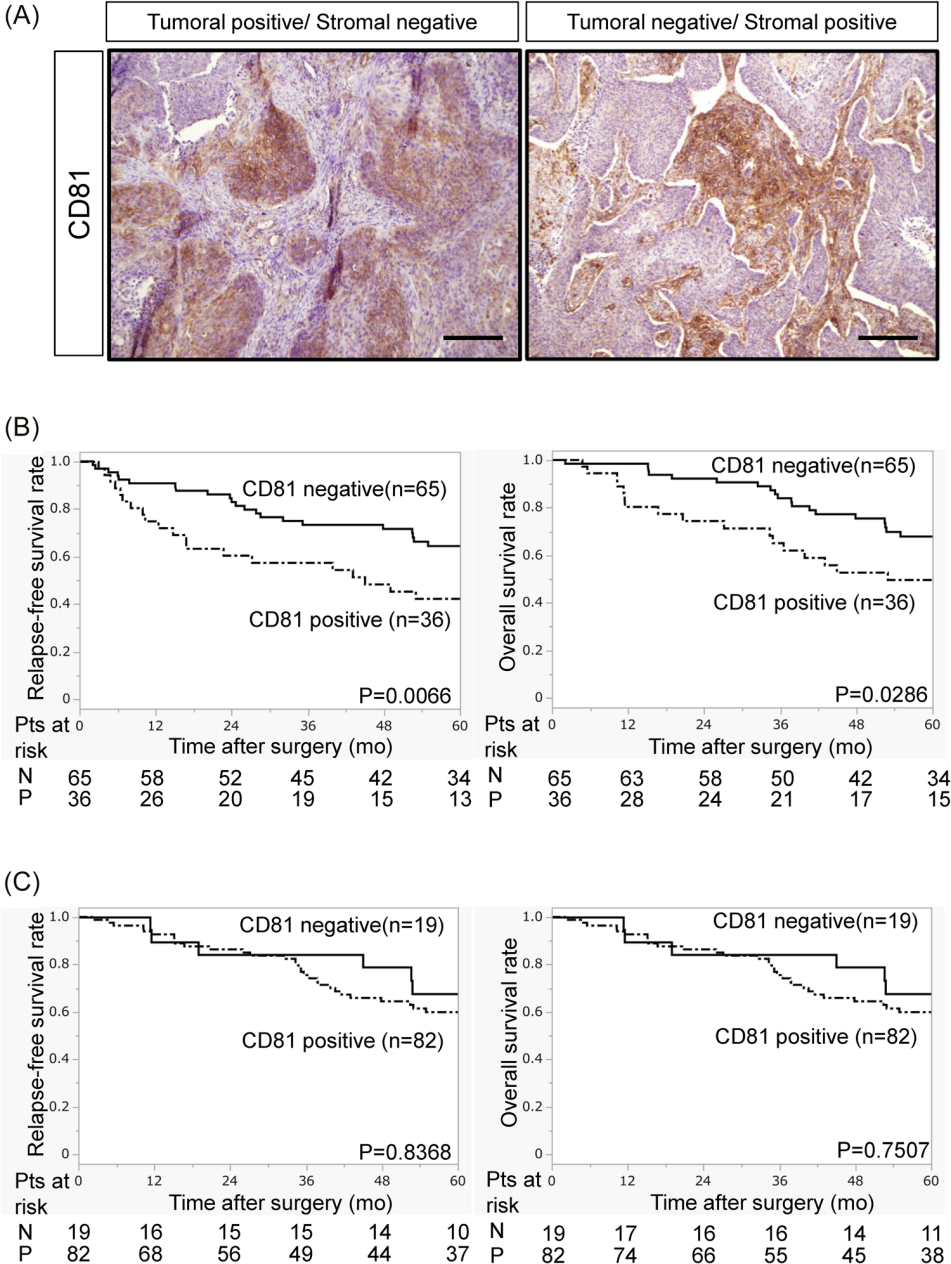
Significance of tumoural and stromal CD81 expression in lung squamous cell carcinoma. (A) Immunohistochemical staining of CD81 in resected lung squamous cell carcinoma tissues. Sections were stained with anti‐CD81 antibody (brown) and counterstained with haematoxylin. Scale bar, 200 µm. Representative images of tumoural CD81‐positive/stromal CD81‐negative tissue and tumoural CD81‐negative/stromal CD81‐positive tissue are shown. (B) Kaplan‒Meier analysis of recurrence‐free survival (RFS) and overall survival (OS) according to tumoural CD81 expression. (C) Kaplan‒Meier analysis of RFS and OS according to stromal CD81 expression.

To evaluate the functional relevance of CD81 in LUSC, we established CD81 knockout (KO) cell lines using CRISPR/Cas9 genome editing in two LUSC models, H520 and HCC15. Loss of CD81 protein was confirmed by Western blotting. These cells were injected subcutaneously into immunodeficient SCID mice. In both models, CD81KO cells formed significantly smaller tumours than wild‐type (WT) controls (Figure 2), indicating that CD81 promotes tumour growth in vivo.

**FIGURE 2:**
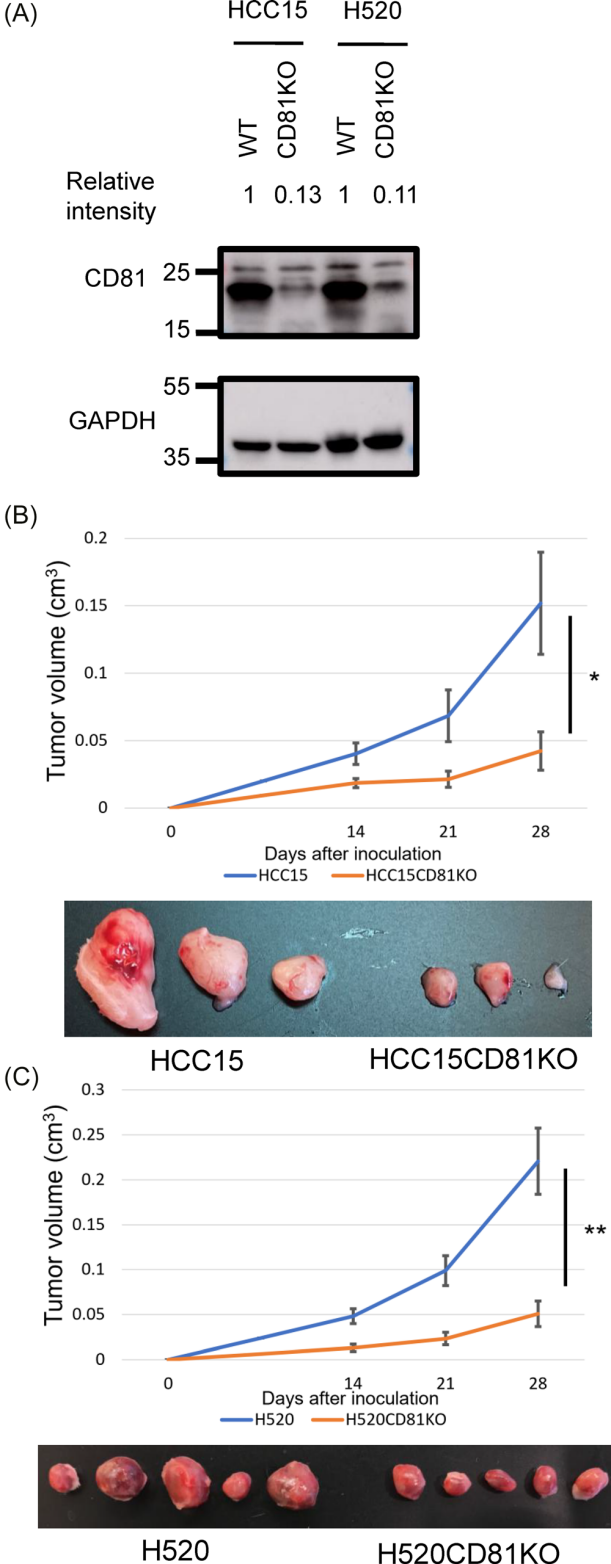
Establishment of CD81 knockout (CD81KO) lung squamous cell carcinoma cell lines and tumour growth in vivo. (A) Western blot analysis of CD81 expression in wild‐type (WT) and CD81KO HCC15 and H520 cells. GAPDH was used as a loading control. (B) (Upper panel) Tumour volume in a murine subcutaneous xenograft model using WT or CD81KO HCC15 cells (pooled analysis of three experiments). (Lower panel) Representative images of excised tumours from each group. (C) (Upper panel) Tumour volume in a murine subcutaneous xenograft model using WT or CD81KO H520 cells. (Lower panel) Representative images of excised tumours from each group.

To investigate mechanisms underlying the tumour‐promoting role of CD81, we performed RNA sequencing of WT and CD81KO HCC15 and H520 cells. Differential gene expression analysis revealed substantial transcriptional alterations in both cell lines (Figure 3A and Tables S4 and S5), with distinct cell‐line‐specific patterns. In H520 cells, CD81KO preferentially affected genes associated with stem‐like properties, extracellular matrix/niche interactions and cellular plasticity, including SPP1, INHBB and HOXA1, suggesting a role for CD81 in regulating stem‐like characteristics.^4^ In contrast, in HCC15 cells, CD81KO predominantly suppressed genes involved in vesicle trafficking, epithelial polarity and cell migration, such as RAB25, MAL2 and LAD1. Notably, IGFBP2, a secreted factor implicated in angiogenesis and tumour invasiveness, was among the most significantly downregulated genes in CD81KO HCC15 cells.^5^ Gene Ontology (GO) enrichment analysis revealed shared functional themes across both cell lines, including pathways related to stemness, migration and angiogenesis (Figure 3B,C and Tables S6 and S7).

**FIGURE 3:**
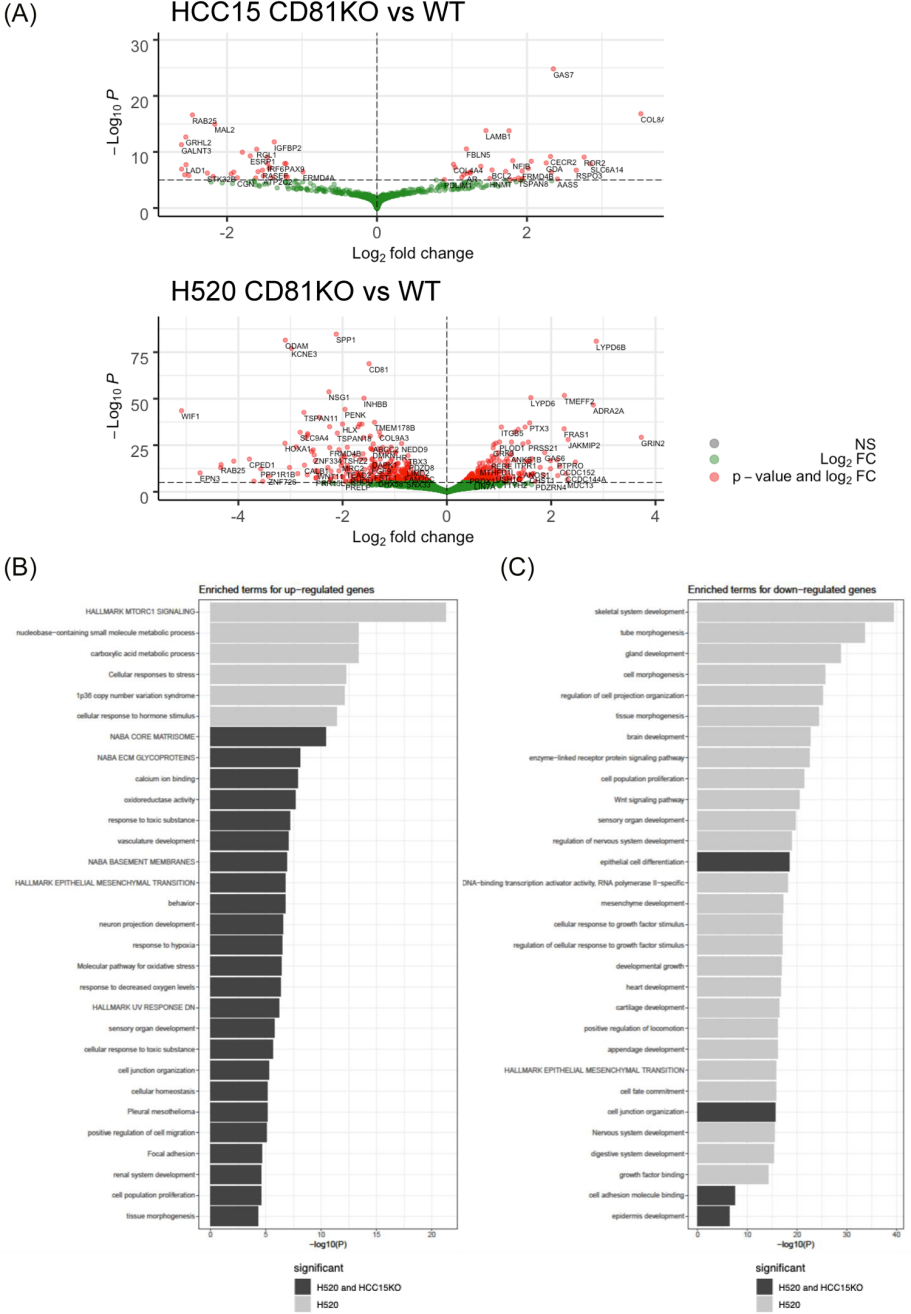
Differential gene expression and pathway enrichment analysis in CD81 knockout (CD81KO) cells. (A) Volcano plots showing differential gene expression between CD81KO and wild‐type (WT) cells in HCC15 and H520 cell lines. (B) Gene Ontology (GO) terms enriched among upregulated genes in CD81KO cells (30 top terms shown). (C) GO terms enriched among downregulated genes in CD81KO cells (30 top terms shown). Only GO terms with adjusted *p*‐value <.01 are displayed. Dark grey indicates GO terms significant in both HCC15 and H520 cells, whereas light grey indicates terms significant only in H520 cells.

Based on the hypotheses generated from the RNA‐sequencing analysis, we next performed functional assays focusing on stemness, migratory capacity and endothelial cell‐mediated angiogenic responses (Figure 4). Using the Aldefluor assay, CD81KO significantly reduced ALDH activity in H520 cells, whereas no significant change was observed in HCC15 cells, indicating cell‐line‐dependent regulation of stem‐like properties. Consistent with this finding, sphere formation was markedly suppressed in CD81KO H520 cells compared with WT cells. In contrast, functional consequences of CD81 loss in HCC15 cells were more prominent in migration and angiogenesis‐related phenotypes. Furthermore, to assess migratory capacity, we performed a scratch wound healing assay, which showed that CD81KO significantly impaired wound closure in HCC15 cells, whereas no significant difference was observed between WT and CD81KO cells in H520 cells. Angiogenic activity was evaluated using an endothelial tube formation assay with HMVEC‐L human lung microvascular endothelial cells exposed to conditioned medium (CM) from WT or CD81KO LUSC cells. CM derived from CD81KO HCC15 cells significantly suppressed endothelial tube formation, as evidenced by reduced junction numbers and tube length (Figure 4D). This suppressive effect was reproducible in MS‐1 murine endothelial cells (Figure S1). In contrast, antibody‐mediated inhibition of IGFBP2 alone did not suppress tube formation (Figure 4D).

**FIGURE 4:**
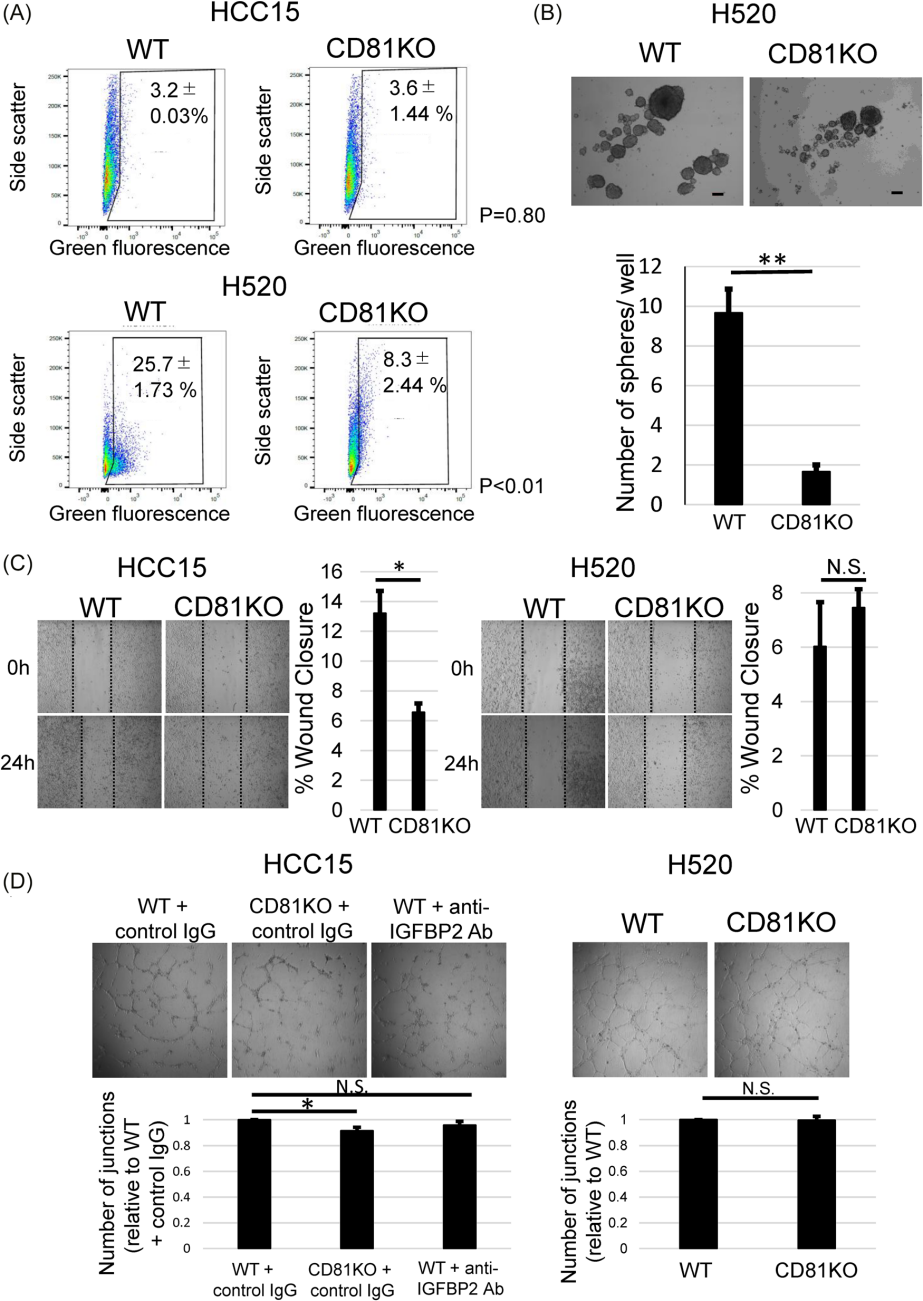
Functional effects of CD81 knockout (CD81KO) on stemness, migration and angiogenesis. (A) Aldefluor assay of wild‐type (WT) and CD81KO HCC15 and H520 cells. Representative flow cytometry plots and quantification of Aldefluor‐positive cells are shown (mean ± SEM, *n* = 3). (B) Sphere formation assay of WT and CD81KO H520 cells. Representative bright‐field images at day 10 (upper panel) and quantification of spheres ≥20 µm (lower panel) are shown. Scale bar, 20 µm. Mean ± SEM of three wells is shown. (C) Scratch wound healing assay in HCC15 and H520 cells. Representative images at 0 and 24 h and quantification of wound closure are shown. Dashed lines indicate wound edges. Data are presented as mean ± SEM. (D) Endothelial tube formation assay using HMVEC‐L cells cultured in conditioned medium (CM) from WT or CD81KO HCC15 or H520 cells. Representative images (upper panels) and quantitative analysis (lower panels) at 6 h after seeding are shown. Data are presented as mean ± SEM.

Taken together, these findings suggest that CD81 contributes to LUSC progression through distinct mechanisms—enhancing stemness in H520 cells and promoting migration and angiogenesis in HCC15 cells—underlining its association with poor prognosis and the importance of accounting for tumour heterogeneity when considering CD81 as a therapeutic target. The reduction in tumour growth observed in CD81KO murine models using two LUSC cell lines is consistent with previous findings by Ye et al., who reported that CD81 knockdown suppressed proliferation in the SK‐MES‐1 cell line.^2^ These results support the tumour‐promoting role of CD81 and its potential as a therapeutic target. CD81 regulates cancer stemness, migration and angiogenesis in a cell‐line‐specific manner, supported by prior findings that it interacts with CD44 to promote tumour cluster formation and metastasis in triple‐negative breast cancer and is enriched in clustered circulating tumour cells with enhanced stem‐like and metastatic properties.^6^ Furthermore, angiogenesis is regulated by interactions between soluble factors and exosome‐mediated signalling, processes in which CD81 has been implicated. Our analyses indicate that inhibition of IGFBP2 alone was insufficient to recapitulate the angiogenic suppression observed in CD81KO cells, suggesting that CD81 regulates angiogenesis through multiple pro‐angiogenic mediators rather than a single dominant factor. Therapies targeting CD81, such as inhibitory peptides and antibodies, are under development,^7^, ^8^ and their potential application in LUSC warrants further investigation.

In conclusion, we demonstrate that CD81 promotes LUSC progression and is linked to poor post‐surgical survival. Its depletion reduces tumour growth, stemness, migration and angiogenesis in preclinical models, highlighting CD81 as a potential prognostic biomarker and therapeutic target.

## AUTHOR CONTRIBUTIONS

*Conceptualisation, data curation, formal analysis, funding acquisition, investigation, methodology, project administration, resources, software, validation, visualisation and writing—original draft*: Ryu Kanzaki. *Methodology, resources and writing—review and editing*: Steven Reid. *Formal analysis, methodology, software, visualisation and writing—review and editing*: Paulina Bolivar. *Data curation*: Sara Larsson. *Resources, funding acquisition and writing—review and editing*: Yasushi Shintani. *Conceptualisation, data curation, funding acquisition, project administration, resources, supervision and writing—review and editing*: Kristian Pietras. The work reported in the paper has been performed by the authors, unless clearly specified in the text.

## CONFLICT OF INTEREST STATEMENT

The authors declare they have no potential conflicts of interest.

## FUNDING INFORMATION

This work was supported by the Swedish Cancer Society (grant number 19 0098 PT) to R.K. and (grant number 21 1808 Pj) to K.P., the Japan Society for the Promotion of Science (grant number 23K15554) to R.K. and (grant number 19H03745) to Y.S., the Swedish Research Council (grant number 2018‐03086), the Göran Gustafsson Foundation, the Cancera Foundation and the Mats Paulsson Foundations to K.P.

## ETHICS STATEMENT

All animal experiments were performed according to the institutional guidelines and approved by the local ethics committee in Lund (permit number 14122‐2020). The human samples were examined following the approval of the Ethical Review Board for Clinical Studies at Osaka University (control number 18518‐6), and the requirement to obtain informed consent was waived.

## Supporting information



Supporting information

Supporting information

Supporting information

Supporting information

Supporting information

Supporting information

Supporting information

## Data Availability

Only publicly available data were used in this study, and data sources and handling of these data are described in the Materials and Methods. Further details and other data that support the findings of this study are available from the corresponding authors upon request.
