# Supplementary material for: CD81 promotes proliferation and predicts survival in lung squamous cell carcinoma
Source: Clin Transl Med. 2026 Apr 20;16(4):e70672. doi: 10.1002/ctm2.70672 (PMC13096717; doi:10.1002/ctm2.70672)
Supplement: Supplementary file 1 — Supporting information [file CTM2-16-e70672-s001.docx]

Supplementary table 1 Patient characteristics according tumoral CD81 positivity.

| Factor | Tumoral CD81 negative (n=65) | Tumoral CD81 positive (n=36) | P value |
| --- | --- | --- | --- |
| Age |  |  |  |
| Range | 42-85 | 38-89 |  |
| Mean ± Standard Deviation | 70.7 ± 7.5 | 71.4 ± 9.9 | 0.6921 |
| Sex |  |  | 0.0918 |
| Male | 56 (86%) | 35 (97%) |  |
| Female | 9 (14%) | 1 (3%) |  |
| Smoking history |  |  | 1.000 |
| Smoker | 62 (95%) | 35 (97%) |  |
| Non-smoker | 3 (5%) | 1 (3%) |  |
| Brinkman index |  |  |  |
| Range | 0-4840 | 0-3500 |  |
| Mean ± Standard Deviation | 1266 ± 766 | 1320 ± 795 | 0.7356 |
| %VC |  |  | 1.000 |
| <80 | 8 (12%) | 4 (11%) |  |
| >=80 | 57 (88%) | 32 (89%) |  |
| %FEV1.0 |  |  | 0.6763 |
| <80 | 27 (42%) | 17 (47%) |  |
| >=80 | 38 (58%) | 19 (53%) |  |
| Preoperative serum CYFRA21-1 level (ng/ml) |  |  | 0.0940 |
| <=2.2 | 41 (63%) | 16 (44%) |  |
| >2.2 | 24 (37%) | 20 (56%) |  |
| Type of resection |  |  | 0.7396 |
| Pneumonectomy | 2 (3%) | 1 (3%) |  |
| Bilobectomy | 1 (2%) | 2 (6%) |  |
| Lobectomy | 48 (74%) | 27 (75%) |  |
| Segmentectomy | 7 (11%) | 4 (11%) |  |
| Partial resection | 7 (11%) | 2 (6%) |  |
| Extent of lymph node dissection |  |  | 0.4013 |
| 0 | 14 (22%) | 6 (17%) |  |
| 1a | 11 (17%) | 8 (22%) |  |
| 1b | 0 (0%) | 1 (3%) |  |
| 2a-1 | 9 (14%) | 2 (6%) |  |
| 2a-2 | 31 (48%) | 19 (53%) |  |
| Completeness of resection |  |  | 0.5366 |
| R0 | 63 (97%) | 36 (100%) |  |
| R1 | 2 (3%) | 0 (0%) |  |
| R2 | 0 (0%) | 0 (0%) |  |
| pT |  |  | 0.1369 |
| 1a | 12 (18%) | 5 (14%) |  |
| 1b | 12 (18%) | 7 (19%) |  |
| 2a | 27 (42%) | 10 (28%) |  |
| 2b | 5 (8%) | 10 (28%) |  |
| 3 | 8 (12%) | 4 (11%) |  |
| 4 | 1 (2%) | 0 (0%) |  |
|  |  |  |  |
| Pathologic stage |  |  | 0.4568 |
| IA | 22 (34%) | 9 (25%) |  |
| IB | 24 (37%) | 10 (28%) |  |
| IIA | 7 (11%) | 8 (22%) |  |
| IIB | 7 (11%) | 5 (14%) |  |
| IIIA | 4 (6%) | 4 (11%) |  |
| IV | 1 (2%) | 0 (0%) |  |
| Postoperative chemotherapy | 6 (9%) | 5 (14%) | 0.5152 |

Supplementary table 2 Univariate and multivariate analysis for factors influencing the RFS after pulmonary resection.

|  | Univariate analysis | | |  | Multivariate analysis | | |
| --- | --- | --- | --- | --- | --- | --- | --- |
| Factors | Hazard ratio | 95%CI | p value |  | Hazard ratio | 95%CI | p value |
| Tumoral CD81 positive/ negative | 2.15 | 1.22-3.78 | 0.008 |  | 2.05 | 1.13-3.70 | 0.018 |
| Stromal CD81 positive/ negative | 0.93 | 0.48-1.83 | 0.8368 |  |  |  |  |
| Age (>70/ <=70) | 1.7 | 0.96-3.03 | 0.0699 |  |  |  |  |
| Sex (Male/ Female) | 1.96 | 0.61-6.31 | 0.2595 |  |  |  |  |
| BI (>1000/ <=1000) | 1.42 | 0.79-2.54 | 0.239 |  |  |  |  |
| %VC (<80/ >=80)) | 2.28 | 1.10-4.1 | 0.0264 |  | 2.1 | 0.96-4.59 | 0.0624 |
| %FEV1.0 (<80/ >=80) | 1.25 | 0.71-2.19 | 0.4384 |  |  |  |  |
| Preoperative serum CYFRA21-1 level (ng/ml) (>2.2/ <=2.2) | 2.13 | 1.21-3.74 | 0.0086 |  | 1.89 | 1.04-3.42 | 0.0358 |
| Type of resection (partial/ anatomical resection) | 3.34 | 1.46-7.62 | 0.0043 |  | 3.6 | 1.52-8.56 | 0.0037 |
| pStage (III,IV/ I,II) | 2.13 | 1.06-4.29 | 0.0336 |  | 1.49 | 0.70-3.17 | 0.3009 |
| Postoperative chemotherapy (yes/ no) | 0.34 | 0.11-1.11 | 0.0742 |  |  |  |  |

Supplementary table 3 Univariate and multivariate analysis for factors influencing the OS after pulmonary resection.

|  | Univariate analysis | | |  | Multivariate analysis | | |
| --- | --- | --- | --- | --- | --- | --- | --- |
| Factors | Hazard ratio | 95%CI | p value |  | Hazard ratio | 95%CI | p value |
| Tumoral CD81 positive/ negative | 1.95 | 1.06-3.59 | 0.0316 |  | 1.95 | 1.03-3.71 | 0.0411 |
| Stromal CD81 positive/ negative | 0.89 | 0.44-1.82 | 0.7509 |  |  |  |  |
| Age (>70/ <=70) | 1.94 | 1.03-3.64 | 0.0389 |  | 1.86 | 0.97 | 0.634 |
| Sex (Male/ Female) | 1.62 | 0.50-5.26 | 0.4183 |  |  |  |  |
| BI (>1000/ <=1000) | 1.22 | 0.66-2.27 | 0.5232 |  |  |  |  |
| %VC (<80/ >=80) | 2.34 | 1.08-5.08 | 0.0312 |  | 2.4 | 1.00-5.72 | 0.049 |
| %FEV1.0 (<80/ >=80) | 1.11 | 0.60-2.03 | 0.746 |  |  |  |  |
| Preoperative serum CYFRA21-1 level (ng/ml) (>2.2/ <=2.2) | 2.35 | 1.28-4.33 | 0.0061 |  | 2.15 | 1.14-4.06 | 0.0177 |
| Type of resection (partial/ anatomical resection) | 2.72 | 1.13-6.60 | 0.0262 |  | 2.23 | 0.83-5.97 | 0.1121 |
| pStage (III,IV/ I,II) | 2.15 | 1.02-4.51 | 0.0432 |  | 1.3 | 0.60-2.82 | 0.5010 |
| Postoperative chemotherapy (yes/ no) | 0.41 | 0.13-1.34 | 0.1407 |  |  |  |  |
