# Supplementary figures and images for: CD81 promotes proliferation and predicts survival in lung squamous cell carcinoma
Source: Clin Transl Med. 2026 Apr 20;16(4):e70672. doi: 10.1002/ctm2.70672 (PMC13096717; doi:10.1002/ctm2.70672)

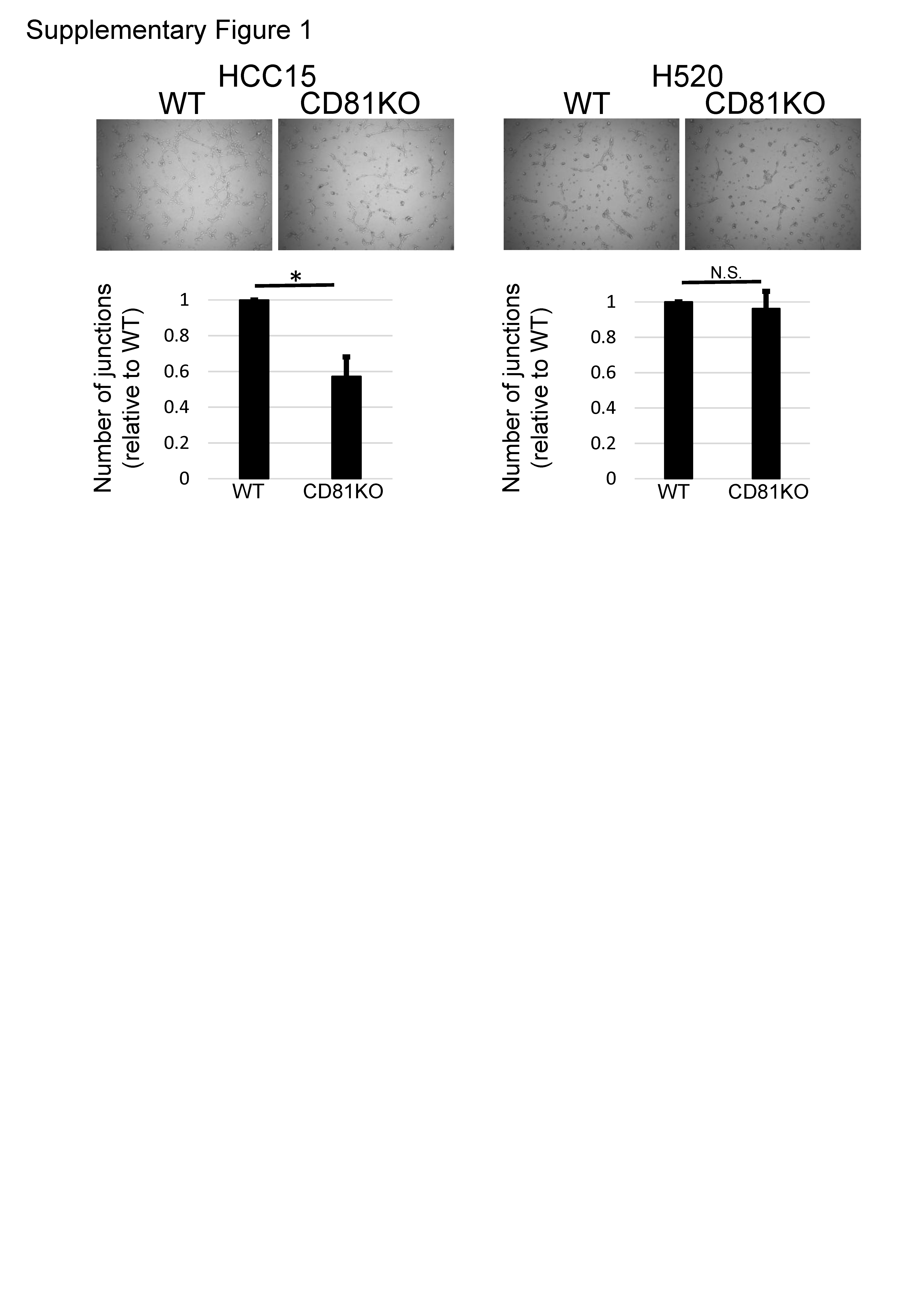

Supplement: Supplementary file 6 — Supporting information [file CTM2-16-e70672-s006.tif]
