# Supplementary material for: CD81 promotes proliferation and predicts survival in lung squamous cell carcinoma
Source: Clin Transl Med. 2026 Apr 20;16(4):e70672. doi: 10.1002/ctm2.70672 (PMC13096717; doi:10.1002/ctm2.70672)
Supplement: Supplementary file 7 — Supporting information [file CTM2-16-e70672-s007.docx]

**Materials and methods**

*Immunohistochemistry*

LUSC was diagnosed by pathologists at Osaka University Hospital and immunohistochemistry was performed as described previously ^1^. Following tumor classification by H&E staining, serial 2-μm paraffin-embedded sections were prepared. The sections were deparaffinized, rehydrated, and incubated in antigen retrieval buffer (pH 6, S1699, Dako, Glostrup, Denmark) for 10 min at 121℃. The sections were then incubated at 4℃ overnight with rabbit polyclonal anti-human CD81 antibody (1/100) (HPA007234, Atlas antibodies, Stockholm, Sweden). HRP-labeled secondary antibody (DakoEnvision Dual link K4003, Dako, Glostrup, Denmark) was applied and incubated at room temperature for 1 h. For visualization, diaminobenzidine (Liquid DAB+ Substrate Chromogen System K3468, Dako, Glostrup, Denmark) was used and the sections were counterstained with hematoxylin. Cancer cells and stromal tissues were identified by hematoxylin staining. Tumoral or stromal CD81 expression was examinmed separately and defined as positive when it was observed in >5% of the tumoral or stromal area.

*Cell culture*

NCI-H520 human LUSC cell line was purchased from the American Type Culture Collection (ATCC, Manassas, VA, USA) and HCC15 human lung squamous cell carcinoma cell line t was purchased from the DSMZ (Braunschweig, Germany). These were maintained in RPMI1640 (Corning Inc., Corning, NY, USA) containing 10% FBS (Gibco, Carlsbad, CA, USA) and penicillin/streptomycin (Corning Inc., Corning, NY, USA). The MS1 murine endothelial cell line was purchased from the American Type Culture Collection (ATCC, Manassas, VA, USA) and maintained in DMEM (Corning Inc., Corning, NY, USA) containing 10% FBS (Gibco, Carlsbad, CA, USA) and penicillin/streptomycin (Corning Inc., Corning, NY, USA).

*Establishment of CD81KO cells*

The sgRNA was designed based on exon 4 of the human CD81 gene and predicted using an online tool developed by Prof. Zhang (http://crispr.mit.edu/). The gRNA sequence GACAAAGCCCCAGATGCCGG was inserted into a lentiCRISPR V2 vector (Addgene), which was kindly provided by Feng Zhang (Addgene plasmid # 52961; http://n2t.net/addgene:52961; RRID:Addgene_52961).^2^ The double-stranded guide sequence oligonucleotides were ligated into lentiCRISPRv2 which was linearized with *BsmB*I restriction enzyme using T4 DNA ligase, and the plasmid was transformed into *Escherichia coli* competent cells. The lentiCRISPRv2 recombinant plasmid along with psPAX2 and pMD2G plasmids were cotransfected into HEK293T, and lentivirus was harvested at 48 h. The lentivirus solution (1 mL) was mixed with 5 μg/ml polybrene and slowly added to NCI-H520 or HCC15 cells cultured in a 6-well plate. The medium was replaced with complete medium after 24 h and the cells were selectively cultured using 1 μg/ml puromycin for 7 days.

*Western blot analysis*

Cell lysates were prepared by lysing the cells in RIPA buffer (89900, Thermo Fisher Scientific, Fremont, CA, USA) and mixing with Laemmli sample buffer (1610747, BioRad, Hercules, CA, USA). Whole cell lysates were separated by SDS-PAGE using 4%–20% Mini-PROTEAN® TGX™ Precast Protein Gels (4561096, BioRad, Hercules, CA, USA). The proteins were then transferred to nitrocellulose membranes (1704158, BioRad, Hercules, CA, USA), blocked, and incubated with rabbit polyclonal anti-human CD81 antibody (1/1000) (ab59477, Abcam, Cambridge, MA, USA) or horseradish peroxidase-conjugated mouse anti-GAPDH antibody (1/2000, 3683S, Cell Signaling Technology, Beverly, MA) according to the manufacturer’s protocol. Proteins were detected with horseradish peroxidase-conjugated anti-rabbit secondary antibody (Cell Signaling Technology, Beverly, MA) and developed with the Immobilon Forte Western HRP substrate (WBLUF0500, Millipore, Milford, MA, USA). The blots were scanned using an Amersham Imager 600 (GE Healthcare Bio-Sciences, Pittsburg, PA, USA).

*Scratch wound healing assay*

Scratch wound healing assays were performed using H520 or H520 CD81 knockout (CD81KO) cells, and HCC15 or HCC15 CD81KO cells. Cells were maintained in RPMI-1640 medium supplemented with 10% FBS and 1% penicillin–streptomycin in a humidified incubator at 37 °C with 5% CO₂. For the assay, 1 × 10⁵ cells were seeded into each well of a 24-well plate in RPMI-1640 medium supplemented with 10% FBS and cultured until they reached approximately 80–90% confluency. The medium was then replaced with RPMI-1640 containing 1% FBS, and cells were serum-starved overnight prior to scratch generation to minimize the effect of cell proliferation on wound closure.

A straight scratch was created through the cell monolayer using a sterile 200-µL pipette tip, and detached cells were gently removed by washing with phosphate-buffered saline (PBS). Fresh RPMI-1640 medium containing 10% FBS was added, and phase-contrast images of the wound area were captured immediately after scratching (0 h) using an inverted Zeiss microscope at ×40 magnification. To ensure consistent imaging of the same wound region over time, scratches were generated as straight and centrally positioned as possible, and the imaging area was marked on the outer edge of each well. Images of the wound area were acquired again 24 h after scratch formation. For each experimental condition, five wells per group were analyzed, selecting wells with clean and uniform scratches. All experiments were independently repeated three times.

*Endothelial cell tube formation*

Endothelial cell tube formation assays were performed using HMVEC-L human lung microvascular endothelial cells (Lonza, Basel, Switzerland). Cells (1 × 10⁴ per well) were seeded into 96-well plates precoated with Cultrex Reduced Growth Factor Basement Membrane Extract (Bio-Techne, Abingdon, UK) in 100 μL of conditioned medium (CM).

Cultrex was thawed slowly at 4 °C overnight and allowed to polymerize at 37 °C for at least 30 min before cell seeding. Conditioned media were obtained from H520 wild-type (WT), H520 CD81 knockout (CD81KO), HCC15 WT, or HCC15 CD81KO cells. Briefly, 6 × 10⁶ H520 or H520 CD81KO cells, or 6 × 10⁵ HCC15 or HCC15 CD81KO cells, were seeded in 10 mL RPMI-1640 medium supplemented with 10% fetal bovine serum in T75 flasks. After 5 days, CM was collected and cleared of cells and debris by centrifugation.

For H520-derived CM, tube formation assays were performed without antibody inhibition, comparing WT-CM and CD81KO-CM. For HCC15-derived CM, antibody inhibition experiments were additionally conducted using HMVEC-L cells only. CM from HCC15 WT cells was incubated with either normal goat IgG control antibody (R&D Systems, AB-108-C) or anti-IGFBP-2 antibody (R&D Systems, AF674; goat polyclonal) at a final concentration of 10 µg/mL prior to cell seeding, while CM from HCC15 CD81 knockout (CD81KO) cells was incubated with normal goat IgG control antibody only. In addition, tube formation assays using MS-1 murine endothelial cells were performed to compare the effects of CM from WT and CD81KO tumor cells without antibody inhibition, serving as a complementary validation of CD81-dependent effects on endothelial tube formation. Endothelial cells were added directly to the CM or CM–antibody mixture and distributed into four replicate wells per condition (one additional well was prepared as backup). Tube formation was assessed 6 h after seeding. One phase-contrast image was captured from the center of each well using an inverted Zeiss microscope at low magnification (×40 objective). Wells showing uneven Cultrex polymerization were excluded from analysis. The number of junctions and total tube length were quantified using ImageJ software with the Angiogenesis Analyzer plugin.^3^ Four wells per group were analyzed, and all experiments were independently repeated at least three times.

*Aldefluor® assay*

ALDH activity was evaluated using the Aldefluor® assay kit (STEMCELL Technologies, Vancouver, BC, Canada) following the guidelines provided by the manufacturer. In brief, dissociated single cells derived from cell lines or spheres were resuspended in Aldefluor® assay buffer containing the ALDH substrate, bodipy-aminoacetaldehyde (BAAA), at a concentration of 7.5 μM, and incubated for one hour at 37°C. An equivalent reaction was conducted in the presence of 15 mM diethylaminobenzaldehyde (DEAB), an inhibitor specific to ALDH. The fluorescence intensity of the stained cells was analyzed using an Flow Cytometer (FACSVerse, BD Biosciences). The DEAB reaction was utilized to establish the baseline for the assay, indicating fluorescence not linked to ALDH activity. The assessment of ALDH activity in a sample was determined by measuring the fluorescence intensity that surpassed the threshold defined by the DEAB reaction.

*Sphere formation assay*

Standard sphere formation assay was performed according to a previous report. ^4^. 5 × 10^3^ H520 or H520CD81KO were seeded in 500µl serum free DMEM/F12 (Corning Inc., Corning, NY, USA) supplemented with B27 (Invitrogen), 20 ng/ml EGF and 20 ng/ml bFGF (Invitrogen) in ultra-low attachment culture plates (Fisher Scientific, Pittsburgh, PA). Cells were cultured for 10 days to form spheres. Number of sphere (>=20µm) was counted by microscope. Each experiment consists of 3 wells and the experiment was repeated 3 times.

*Animal experiments*

All animal experiments were performed according to the institutional guidelines and approved by the local ethics committee in Lund (permit number 14122-2020). SCID female mice (6-7 weeks of age) were purchased from Scanbur (Karlslunde, Denmark).

The experiments were repeated 4 times. Subcutaneous inoculation was performed by injecting 2x10^6^ H520 or H520 CD81KO cells or HCC15 or 5x10^6^ HCC15 CD81KO cells into the flanks of the mice. Tumor volumes were measured with calipers and calculated as width x width x length x 0.52. The experiment was repeated 3 times.

*RNA-seq data processing and analysis*

WT or CD81KO HCC15 or H520 cells were seeded in 6-well plates and RNA was isolated from subconfluent state. RNA was isolated from 3 times from different cultures. Cells were lysed in RLT buffer and passed through a QiaShredder (Qiagen #79656) before RNA was isolated using the RNeasy kit (Qiagen #74106). RNA sequencing and processing was performed by the Center for Translational Genomics, Lund University. Briefly, library preparation was performed using TruSeq Stranded mRNA Library Prep (20020594, Illumina) and the raw sequencing data was generated using the NovaSeq 6000 System (20012850, Illumina). Demultiplexing raw data to FASTQ files was performed using bcl2fastq (Illumina) followed by a quality assessment of the FASTQ files using FastQC ^5^. Alignment of reads was performed using the STAR software ^6^. The reference genome sequence was retrieved from the Ensembl database release ^7^, the Human GRCh38 primary assembly, and the annotation (GTF) from gencode version 33 ^8^. Quantification of the expression levels of each gene was performed using the featureCounts software ^9^, with the following settings: paired-end mode (-p), strand specific (-s 2), no multimapping reads counted, counting exonic reads. Differential gene expression analysis between CD81KO HCC15 and WT cells was performed using Deseq2 (v1.26) ^10^. For gene ontology (GO) analysis, an overrepresentation analysis was performed using metascape (v3.5.20250701). We uploaded our DGE results as two different multiple gene list (one for up-regulated genes and one for down-regulated genes of both cell lines together). We selected GO Molecular Functions, GO Biological Processes, BioCarta Gene Sets, Hallmark Gene Sets, Reactome Gene Sets, KEGG Pathway, WikiPathways, Canonical Pathways and PANTHER Pathways for the enrichment analysis ^11^.

*Patients and specimens*

Between 2007 and December 2014, 118 patients with lung squamous cell carcinoma underwent surgery at Osaka University Hospital. After excluding 6 patients who underwent preoperative therapy and 11 patients whose samples were not available, 101 patients were included in the present study. Staging, extent of lymph node dissection, and completeness of resection was assessed based on the general rules for the clinical and pathological recording of lung cancer from the Japan Lung Cancer Society, 7^th^ edition. ^12^ The median follow-up period was 55 months (range 2–119). The samples were examined following the approval of the Ethical Review Board for Clinical Studies at Osaka University (control number 18518-6).

*Statistical analyses*

Statistical analyses were performed using the JMP Pro 17.0 software program (SAS Institute, Berkley, CA, USA). The data are expressed as the mean ± standard deviation or median values. Student's t-test and Fisher's exact test were used to compare the two groups. The relapse-free survival (RFS), overall survival (OS) rates after pulmonary resection were analyzed using the Kaplan-Meier method. The RFS was defined as the time interval between the date of pulmonary resection and relapse or death or the last follow-up for live patients. The OS was defined as the time interval between the date of pulmonary resection and death or the last follow-up for live patients. The Cox constant proportional hazards model was used to assess the effects of the covariates on the RFS and OS. Statistical significance was set at P <0.05. Factors with P values <0.05 in the univariate analysis were used for the subsequent multivariate analysis.

References

1. Kanzaki R, Reid S, Bolivar P, et al. FHL2 expression by cancer-associated fibroblasts promotes metastasis and angiogenesis in lung adenocarcinoma. *International journal of cancer Journal international du cancer*. Sep 8 2024;doi:10.1002/ijc.35174

2. Sanjana NE, Shalem O, Zhang F. Improved vectors and genome-wide libraries for CRISPR screening. *Nat Methods*. Aug 2014;11(8):783-784. doi:10.1038/nmeth.3047

3. Carpentier G, Berndt S, Ferratge S, et al. Angiogenesis Analyzer for ImageJ - A comparative morphometric analysis of "Endothelial Tube Formation Assay" and "Fibrin Bead Assay". *Scientific reports*. Jul 14 2020;10(1):11568. doi:10.1038/s41598-020-67289-8

4. Berens EB, Holy JM, Riegel AT, Wellstein A. A Cancer Cell Spheroid Assay to Assess Invasion in a 3D Setting. *Journal of visualized experiments : JoVE*. Nov 20 2015;(105)doi:10.3791/53409

5. S. A. FastQC: a quality control tool for high throughput sequence data. *Babraham Bioinformatics*. 2010;Babraham Institute, Cambridge, United Kingdom

6. Dobin A, Davis CA, Schlesinger F, et al. STAR: ultrafast universal RNA-seq aligner. *Bioinformatics*. Jan 1 2013;29(1):15-21. doi:10.1093/bioinformatics/bts635

7. Dyer SC, Austine-Orimoloye O, Azov AG, et al. Ensembl 2025. *Nucleic acids research*. Jan 6 2025;53(D1):D948-d957. doi:10.1093/nar/gkae1071

8. gencode version 33.https://www.gencodegenes.org/human/release_33.html.

9. Liao Y, Smyth GK, Shi W. featureCounts: an efficient general purpose program for assigning sequence reads to genomic features. *Bioinformatics*. Apr 1 2014;30(7):923-30. doi:10.1093/bioinformatics/btt656

10. Love MI, Huber W, Anders S. Moderated estimation of fold change and dispersion for RNA-seq data with DESeq2. *Genome biology*. 2014;15(12):550. doi:10.1186/s13059-014-0550-8

11. Zhou Y, Zhou B, Pache L, et al. Metascape provides a biologist-oriented resource for the analysis of systems-level datasets. *Nature communications*. Apr 3 2019;10(1):1523. doi:10.1038/s41467-019-09234-6

12. Society JLC. *general rules for lung cancer*

2010.

Supplementary Figure Legend

Supplementary Figure 1

Results of an endothelial cell tube formation assay. Murine endothelial MS1 cells were seeded in conditioned medium from WT and CD81KO - HCC15 and H520 cells. Photographs taken 6 h post-seeding were analyzed. (Upper panels) Representative photographs of WT and KO groups are shown. (Lower panels) results of the quantification of tube formation are shown. Data represent the mean ± SEM.
